# Quantitative bacterial counts in the bone marrow of Vietnamese patients with typhoid fever

**DOI:** 10.1093/trstmh/trac003

**Published:** 2022-01-29

**Authors:** Pham Van Be Bay, John Wain, Le Thi Phuong, Vo Anh Ho, Tran Tinh Hien, Christopher M Parry

**Affiliations:** Dong Thap Provincial Hospital, Cao Lanh, Dong Thap Province, Vietnam; Wellcome Trust Major Overseas Programme, Oxford University Clinical Research Unit, Hospital for Tropical Diseases, 764 Vo Van Kiet, District 5, Ho Chi Minh City, Vietnam; Quadram Institute Bioscience, Norwich Research Park, Norwich, UK; Dong Thap Provincial Hospital, Cao Lanh, Dong Thap Province, Vietnam; Dong Thap Provincial Hospital, Cao Lanh, Dong Thap Province, Vietnam; Well come Trust Major Overseas Programme, Oxford University Clinical Research Unit, Hospital for Tropical Diseases, 764 Vo Van Kiet, District 5, Ho Chi Minh City, Vietnam; Wellcome Trust Major Overseas Programme, Oxford University Clinical Research Unit, Hospital for Tropical Diseases, 764 Vo Van Kiet, District 5, Ho Chi Minh City, Vietnam; Department of Clinical Sciences, Liverpool School of Tropical Medicine, Liverpool, UK; Centre for Tropical Medicine and Global Health, Nuffield Department of Clinical Medicine, University of Oxford, New Richards Building, Old Road Campus, Roosevelt Drive, Oxford, OX3 7LG, UK

**Keywords:** azithromycin, ofloxacin, multiple drug resistance, quantitative bone marrow counts, *Salmonella enterica* serovar typhi, typhoid fever

## Abstract

**Background:**

Bone marrow culture (BMC) is the reference standard for typhoid fever diagnosis. We studied the additional yield of BMC over blood culture (BC) and the relationship between quantitative BMC counts and severe disease.

**Methods:**

Hospitalised Vietnamese patients with suspected typhoid fever were prospectively investigated with a BC, BMC, faecal culture and quantitative BMC counts.

**Results:**

*Salmonella* typhi was isolated in 195 of 231 patients: from BC and BMC in 144 (73.8%), from BMC alone in 33 (16.9%), from BC alone in 12 (6.2%) and from faeces alone in 6 (3.1%). In 167 patients the median extracellular count of *S.* typhi was 2.5 cfu/mL (interquartile range [IQR] 0–10) and the intracellular count was 10.5 cfu/mL (IQR 2–42) with a ratio of 1.3 bacteria/cell (IQR 0.6–2.5). The median count of intracellular bacteria in 24 patients with severe disease was 46 bacteria/cell (IQR 9–105) compared with 6.5 bacteria/cell (IQR 2–34) in 143 with non-severe disease (p=0.005). The intracellular BMC count was negatively correlated with the peripheral white cell count and positively correlated with hepatomegaly, splenomegaly, aspartate transaminase, a positive BC and the fever clearance time following treatment with azithromycin, ofloxacin or a combination of the two.

**Conclusions:**

BMC gave a moderate additional yield over BC. Intracellular BMC counts may reflect the bacterial load in typhoid fever.

## Introduction

*Salmonella enterica* serovar typhi (*Salmonella* typhi) and *Salmonella* paratyphi A are Gram-negative bacteria that cause typhoid and paratyphoid (enteric) fever.^1^ Blood culture is commonly used to confirm the diagnosis of typhoid fever with a sensitivity of 40–80%.^2,3^ Bone marrow aspirate culture (BMC) is more sensitive than blood and is often considered the reference standard, but it is infrequently performed because it is relatively invasive and technically demanding.^2^ BMC may remain positive after starting antimicrobial treatment as the blood culture becomes negative.^4^ The relative yield of BMC compared with that of blood culture varies in different reports.^4–13^ In a systematic review, the sensitivity of blood culture in 529 true-positive cases was 61% (95% confidence interval [CI] 52 to 70) compared with a BMC sensitivity of 96% (95% CI 92 to 99).^14^

The bacterial counts in the blood are low in typhoid fever, invariably <10 colony-forming units (cfu)/mL of blood.^15–19^ In one study of 375 Vietnamese patients with uncomplicated typhoid fever the median number of bacteria was 1 cfu/mL (IQR <0.3–5).^18^ The blood bacterial counts in typhoid are comparable to those in other Gram-negative bloodstream infections despite the different clinical picture of typhoid fever and Gram-negative sepsis.^20^ When treated with adequate antimicrobials, the mortality in hospitalised cases of typhoid fever is 2–5% compared with 20–30% in other types of Gram-negative sepsis.^21,22^ A severe and fatal outcome in other Gram-negative bacteraemias has been associated with high counts in the blood,^23,24^ but the relationship between the development of severe disease and blood bacterial counts has not been fully explored in typhoid fever.^16,18^ The bacterial counts in bone marrow are on average 10-fold higher than in blood in typhoid fever and the organisms are frequently intracellular.^25^ In a study in Indonesia, BMC was positive in 90% of 29 typhoid patients with severe and fatal disease, 75% in 90 severe typhoid patients who survived and 56% in 273 patients with non-severe typhoid.^26^ We hypothesise that the bacterial load in typhoid fever is reflected in the bone marrow bacterial counts and that there is a relationship between bone marrow counts and severe disease and response to antimicrobial treatment.

In this study we report the additional yield of bone marrow compared with blood culture for diagnosis in patients with suspected typhoid fever and the association of bacterial counts in bone marrow with clinical and laboratory parameters, including disease severity.

## Methods

### Patients studied

Patients at Dong Thap Provincial Hospital (DTPH) in Cao Lanh, Dong Thap Province, Vietnam, a 400-bed provincial hospital serving the population of Cao Lanh and a referral centre for the province of Dong Thap in the Mekong Delta, were assessed for potential recruitment for a randomised clinical trial of antimicrobial treatment in typhoid fever.^27^ The study compared a 7-d regimen of azithromycin or ofloxacin or an ofloxacin and azithromycin combination in those considered to have non-severe disease. Patients with severe disease at presentation were treated with a ceftriaxone and azithromycin combination outside of the trial. The study received approval from the scientific and ethical committee of the Dong Thap Provincial Hospital and the Hospital for Tropical Diseases, Ho Chi Minh City, Vietnam. The studies were conducted in accordance with International Conference on Harmonization and the Declaration of Helsinki guidelines. Patients, or the parent or guardian for children, gave fully informed verbal consent before entry into the study.

### Clinical methods

Patients admitted to the hospital infection ward with clinically suspected typhoid fever were eligible. The features suggesting clinical typhoid fever included any (but not necessarily all) of the following: a febrile illness with a duration >3 d, the presence of abdominal symptoms (abdominal pain, diarrhoea or constipation), a documented fever ≥39°C, hepatomegaly and/or splenomegaly, withdrawn or apathetic behaviour, gastrointestinal bleeding, a low or normal white cell count, elevation of liver enzymes (aspartate aminotransferase [AST], alanine aminotransferase [ALT]) 2–3 times above the normal range, no other obvious focus of infection and, where relevant, a negative malaria blood smear. Demographic, clinical and laboratory data were prospectively gathered on standardised case report forms. Clinical outcomes were recorded for all patients. Patients who were readmitted as relapse cases were only included for the initial admission. The fever clearance time was defined as the time from the start of treatment until the body temperature reached ≤37.5°C and remained ≤37.5°C for 48 h.

Severe disease was defined by the presence of one or more of the following features: gastrointestinal bleeding (the presence of visible blood or melaena in the stool), intestinal perforation (confirmed at surgery), encephalopathy (delirium, obtundation or coma), haemodynamic shock (systolic blood pressure <90 mmHg and/or diastolic blood pressure <60 mmHg associated with tissue hypoperfusion), myocarditis (tachycardia or bradycardia with an associated abnormality of the electrocardiogram or ultrasound evidence of a pericardial effusion), hepatitis (as indicated by jaundice and/or hepatomegaly with abnormal levels of AST [>400 IU/L] and/or ALT [>400 IU/L]), a clinical diagnosis of cholecystitis (right upper quadrant pain and tenderness without evidence of hepatitis), pneumonia (respiratory symptoms with abnormal chest radiograph infiltrates) or pleural effusion, the need for a blood transfusion or death in the hospital.^28^ A relapse was defined as recurrence of symptoms and signs suggestive of typhoid fever within the 4-week period after the patient had been discharged well from the hospital accompanied by a blood culture positive for *Salmonella* typhi or *Salmonella* paratyphi A.

### Laboratory methods

Before antimicrobial treatment was started, blood was taken for culture and routine laboratory tests. Two to five millilitres of blood (volume depending on age) for culture was added to 50 mL of brain heart infusion (BHI) broth (all media in the study was from Unipath, Basingstoke, UK) containing sodium polyethanolsulphonate (SPS; 0.05%) and incubated at 35–37°C for 7 d. Subcultures onto sheep blood agar were performed after 1, 2 and 7 d or when the broth went turbid. Stool cultures were performed after enrichment of a 1-g sample in 10 mL of selenite broth for 24 h. Five microlitres from the top of the broth was plated onto XLD agar. Suspect colonies were identified as described below.

A bone marrow aspirate was performed usually within 24–48 h of the blood culture. Bone marrow was aspirated from the superior iliac crest after infiltration of the site with local anaesthetic. The aspirate was inoculated into BHI broth containing SPS (1:10) and processed non-quantitatively in the same way as blood cultures. If there was sufficient sample, the bone marrow aspirate was also cultured by a quantitative method. For a quantitative count, the extra bone marrow aspirate was divided into three equal aliquots. One aliquot was added to 19 mL of molten Columbia agar (±0.05% SPS) in a sterile petri dish, gently mixed and allowed to set. After 2–4 d of incubation at 35–37°C, the number of colonies that had grown in the agar were counted. A straight wire was used to pick up to five colonies to the surface of the agar for standard identification. The number of colony forming units per millilitre of bone marrow sample was calculated. If no colonies were visible after 4 d of incubation the plates were considered negative. The other two aliquots were incubated with 100 μg/mL of gentamicin for 30 min at 37°C to remove extracellular bacteria or were incubated with 0.1% digitonin for 12 min to release intracellular bacteria and then processed by pour plate to determine the quantitative count, as described above.

*Salmonella* isolates were identified by standard biochemical tests and agglutination with *Salmonella*-specific antisera (Murex Diagnostics, Dartford, UK). Antimicrobial susceptibilities were performed at the time of isolation by a modified Bauer–Kirby disc diffusion method and inhibition zone sizes were recorded. Interpretations of the zone sizes were re-evaluated based on the 2019 Clinical and Laboratory Standards Institute (CLSI) guidelines.^29^ The antimicrobials tested were chloramphenicol (30 μg), ampicillin (10 μg), trimethoprim-sulphamethoxazole (1.25/23.75 μg), ceftriaxone (30 μg), ofloxacin (5 μg) and ciprofloxacin (5 μg). Isolates were stored in Protect beads (Prolabs, Oxford, UK) at −20°C and later subcultured for the minimum inhibitory concentration (MIC) by the agar plate dilution method according to CLSI guidelines). Antimicrobial powders were purchased from Sigma-Aldrich (Dorset, UK). *Escherichia coli* ATCC 25922 and *Staphylococcus aureus* ATCC 25923 were used as control strains for these assays. An isolate was defined as multidrug resistant (MDR) if it was resistant to chloramphenicol (≥32 μg/ml), trimethoprim/sulphamethoxazole (≥8/152 μg/ml) and ampicillin (≥32 μg/ml). An isolate was defined as ciprofloxacin non-susceptible if it had a ciprofloxacin MIC >0.06 μg/ml.

### Analysis

The bone marrow bacterial counts in each aliquot were used to derive the number of intracellular and extracellular bacteria present and the average number of intracellular bacteria per cell according to the following formulae:

- Untreated bone marrow aspirate count−gentamicin-treated bone marrow aspirate count=number of extracellular bacteria
- Lysed bone marrow aspirate count−extracellular count=number of intracellular bacteria
- (Lysed bone marrow aspirate count−extracellular count)/(untreated bone marrow aspirate count−extracellular count)=proportion of intracellular bacteria/cell.

If there were no bacteria in the gentamicin-treated bone marrow aspirate there was assumed to be no intracellular bacteria. If the untreated bone marrow aspirate count was higher than the lysed bone marrow aspirate count, it was not possible to determine the number of bacteria/cell. The minimum total amount of bone marrow aspirate cultured was 1 mL, giving a lower limit of detection of 1 cfu/mL. A nominal lower value of 0.2 cfu/mL was assigned when broth bone marrow aspirate cultures were positive but quantitative cultures negative.

Because the continuous measures followed non-normal distributions, continuous data were described using median and IQR and ranges and the Wilcoxon rank sum test or Kruskal–Wallis test was used for within-group comparisons and Spearman's rank correlation for tests of association. Categorical variables were described using numbers and percentages and compared with the χ^2^ test or the Fisher's exact test as appropriate. The association of the extracellular and intracellular quantitative bone marrow aspirate counts with severe typhoid fever was examined in a multivariate logistic regression model including *a priori* factors previously associated with severity: age, sex, duration of illness prior to admission to the hospital, the presence of an MDR phenotype and ciprofloxacin non-susceptible. Statistical analysis was undertaken using Stata/IC 14.2 (StataCorp, College Station, TX, USA).

## Results

### Demographic data and diagnostic test results

A total of 243 febrile Vietnamese children and adults were evaluated for possible typhoid fever. The median age was 9 y (IQR 6–13, range 3–46) and 121 (49.6%) were male. A blood culture was performed in all 243 patients, a BMC in 231 (95.1%) and a faecal culture in 181 (74.5%). *Salmonella* typhi was isolated from blood and/or bone marrow aspirate and/or faeces in 207 (85.2%) patients. *Salmonella* paratyphi A was isolated from the blood culture in one patient, but with a negative BMC. In the 231 patients who had both blood and bone marrow cultures performed, *Salmonella* typhi was isolated from blood, bone marrow and/or faeces on 195 (84.4%) occasions: from both blood and bone marrow aspirate in 144 (73.8%), from bone marrow aspirate alone in 33 (16.9%), from blood culture alone in 12 (6.2%) and from faeces alone in 6 (3.1%) (Table 1). The proportion of patients with a positive blood culture decreased as the duration of illness before admission increased, from 107/124 (86.3%) in week 1 to 10/18 (55.5%) after week 2, whereas the proportion of patients with a positive bone marrow aspirate was 113/124 (91.1%) and 17/18 (94.4%), respectively. The proportion of patients in which the bone marrow aspirate was the sole positive sample increased from 14/124 (11.3%) in week 1 to 7/18 (38.9%) after week 2. A faecal sample was cultured in 178/231 (77.1%) patients who had both blood and bone marrow cultures performed and was positive in 46 (25.8%). On six occasions *S.* typhi was isolated from a faecal sample when the blood and bone marrow culture were both negative.

**Table 1.** Results of blood, bone marrow aspirate and faecal cultures in 195 patients with suspected typhoid fever who had both a blood culture and bone marrow aspirate culture performed with *S*. typhi isolated from at least one site

Duration of symptoms (week)	Number	BC	BMC	Faeces	BC+BMC	BMC alone	BC alone	Faeces alone
1	124	107 (86.3)	113 (91.1)	25/115 (21.7)	99 (79.8)	14 (11.3)	8 (6.5)	3 (2.4)
2	53	39 (73.6)	47 (88.7)	17/46 (36.9)	35 (66.0)	12 (22.6)	4 (7.5)	2 (3.8)
>2	18	10 (55.5)	17 (94.4)	4/17 (23.5)	10 (55.0)	7 (38.9)	0 (0)	1 (5.6)
Total	195	156 (80.0)	177 (90.8)	46 (23.6)	144 (73.8)	33 (16.9)	12 (6.2)	6 (3.1)

Values are presented as n (%).

### Clinical and microbiological features in patients who had a quantitative BMC

There was sufficient bone marrow aspirate to perform a quantitative bone marrow count in 207 patients. *S.* typhi was isolated from blood and/or bone marrow aspirate cultures in 167 (80.7%) of these patients, who had a median age of 7 y (IQR 5–10, range 4–42). The median number of bacteria was 11 cfu/mL (IQR 3–33) in untreated bone marrow aspirate, 4 cfu/mL (IQR 1–22) in the gentamicin-treated sample and 14 cfu/mL (IQR 4–55) in the lysed sample. The calculated intracellular count of bacteria was a median of 10.5 cfu/mL (IQR 2–42) with another 2.5 cfu/mL (IQR 0–10) that were extracellular and a calculated median value for the number of bacteria per cell of 1.3 (IQR 0.6–2.5).

The clinical features, laboratory results and quantitative bone marrow aspirate counts of patients at the time of admission categorised by age ranges are summarised in Table 2. There were no significant differences between the different ages except that children were less likely than adults to have a headache and more likely to have hepatomegaly and a lower haematocrit. There was a trend for quantitative bone marrow aspirate counts to be higher in the younger age group (<10 y). Comparisons according to the duration of symptoms prior to admission are presented in Table 3. Constipation becomes more common and vomiting less common as the duration of symptoms lengthens. There was a trend for the quantitative bone marrow aspirate counts to be higher in the first week compared with later weeks.

**Table 2.** The demographics and admission clinical features, laboratory results and quantitative bone marrow aspirate counts of 167 blood and/or bone marrow aspirate culture–confirmed typhoid fever patients who had a quantitative bone marrow aspirate bacterial count performed, categorised by age

Covariate	All ages (N=167)	4–9 y (n=88)	10–15 y (n=59)	>15 y (n=20)	p-Value^a^
Male, n (%)	73 (44)	40 (46)	25 (42)	8 (40)	0.880
Days ill prior to admission, median (IQR)	7 (5–10)	7 (5–10)	7 (5–10)	7 (6–11)	0.692
Signs and symptoms, n (%)					
Abdominal pain	119 (71)	60 (68)	48 (81)	11 (55)	0.050
Constipation	107 (64)	60 (68)	38 (64)	9 (45)	0.162
Diarrhoea	137 (82)	66 (75)	53 (90)	18 (90)	0.058
Vomiting	79 (47)	38 (43)	29 (57)	12 (60)	0.353
Cough	52 (31)	25 (28)	20 (34)	7 (35)	0.745
Headache	103 (62)	42 (48)	45 (76)	16 (80)	<0.001
Temperature ≥40°C	67 (40)	33 (38)	25 (42)	9 (45)	0.769
Hepatomegaly	107 (64)	67 (76)	34 (58)	6 (30)	<0.001
Splenomegaly	13 (8)	9 (10)	4 (7)	0 (0)	0.356
Laboratory, median (IQR)					
Haematocrit (%)	34 (31–36)	33 (30–35)	34 (32–36)	36 (34–39)	<0.001
White cell count (×10^9^/L)	6.5 (5.4–8.2)	6.5 (5.4–8.1)	7.0 (5.8–8.5)	6.3 (4.7–8.1)	0.355
Neutrophil count (×10^9^/L)	4.3 (3.3–5.4)	4.1 (3.0–5.3)	4.6 (3.7–5.6)	4.2 (2.9–5.2)	0.104
Lymphocyte count (×10^9^/L)	1.9 (1.2–2.6)	2.0 (1.5–2.7)	1.8 (1.2–2.4)	1.7 (0.9–2.2)	0.080
Platelet count (×10^9^/L)	176 (138–247)	182 (131–262)	176 (143–247)	149 (109–176)	0.043
AST (IU/L)	116 (78–210)	131 (80–264)	110 (78–191)	98 (71–129)	0.172
ALT (IU/L)	76 (51–135)	81 (55–140)	73 (50–141)	70 (44–103)	0.431
Microbiology, n (%)					
Fully susceptible organism	15 (9)	8 (9)	5 (9)	2 (10)	1.000
MDR	149 (89)	78 (89)	53 (88)	18 (90)	1.000
Ciprofloxacin non-susceptible	154 (92)	82 (93)	55 (93)	17 (86)	0.426
Faecal culture positive	38/158 (24)	18/82 (22)	14/56 (25)	6/20 (30)	0.702
Quantitative bone marrow counts, median (IQR)					
Untreated bone marrow (cfu/mL)	11 (3–33)	16 (4–49)	5 (1–31)	10 (5–43)	0.030
Gentamicin treated bone marrow (cfu/mL)	4 (1–22)	7 (1–25)	4 (0–17)	3 (2–24)	0.289
Lysed bone marrow (cfu/mL)	14 (4–55)	28 (6–66)	8 (1–37)	11 (6–62)	0.014
Extracellular (cfu/mL)	2.5 (0–10)	3.5 (1–13)	2.0 (0–6)	2.5 (1–14)	0.096
Intracellular (cfu/mL)	10.5 (2–42)	15.0 (2–54)	5.0 (1–30)	5.0 (2–38)	0.073
Proportion of bacteria/cell	1.3 (0.6–2.5)	1.6 (0.8–3.1)	1.3 (0.2–2.0)	1.1 (0.9–1.9)	0.209
Proportion of bacteria intracellular (%)	80.8	81.1	71.4	66.7	

a Comparison of different age groups by χ^2^, Fisher's exact or Mann-Whitney U test as appropriate.

**Table 3.** The demographics and admission clinical features, laboratory results and quantitative bone marrow aspirate counts of 167 blood and/or bone marrow aspirate culture–confirmed typhoid fever patients who had a quantitative bone marrow aspirate bacterial count performed, categorised by week of illness

Covariate	All ages (N=167)	Week 1 (n=106)	Week 2 (n=46)	>Week 2 (n=15)	p-Value^a^
Male, n (%)	73 (44)	48 (45)	19 (41)	6 (40)	0.859
Age (years), median (IQR)	9 (6–12)	10 (7–12)	7 (6–12)	10 (7–14)	0.180
Signs and symptoms, n (%)					
Abdominal pain	119 (71)	72 (68)	35 (76)	12 (80)	0.500
Constipation	107 (64)	63 (59)	30 (65)	14 (93)	0.028
Diarrhoea	137 (82)	85 (80)	39 (85)	13 (87)	0.776
Vomiting	79 (47)	57 (54)	19 (41)	3 (20)	0.032
Cough	52 (31)	27 (25)	20 (44)	5 (33)	0.086
Headache	103 (62)	63 (59)	27 (59)	13 (87)	0.112
Temperature ≥40°C	67 (40)	44 (42)	18 (39)	5 (33)	0.832
Hepatomegaly	107 (64)	65 (61)	32 (70)	10 (67)	0.637
Splenomegaly	13 (8)	7 (7)	4 (9)	2 (13)	0.491
Laboratory, median (IQR)					
Haematocrit (%)	34 (31–36)	34 (32–36)	33 (30–35)	32 (31–37)	0.122
White cell count (×10^9^/L)	6.5 (5.4–8.2)	6.4 (5.4–8.0)	7.2 (5.8–9.0)	6.7 (5.8–8.1)	0.190
Platelet count (×10^9^/L)	176 (138–247)	167 (136–228)	190 (142–293)	180 (138–263)	0.396
Neutrophil count (×10^9^/L)	4.3 (3.3–5.4)	4.2 (3.2–5.3)	4.8 (3.6–5.9)	4.4 (3.5–5.1)	0.406
Lymphocyte count (×10^9^/L)	1.9 (1.2–2.6)	1.8 (1.2–2.4)	2.0 (1.5–2.6)	2.3 (1.6–2.6)	0.256
AST (IU/L)	116 (78–210)	122 (80–231)	143 (82–264)	91 (72–141)	0.276
ALT (IU/L)	76 (51–135)	76 (51–131)	75 (55–166)	73 (51–150)	0.395
Microbiology, n (%)					
Fully susceptible organism	15 (9.0)	8 (8)	6 (13)	1 (7)	0.489
MDR	149 (89.2)	96 (91)	39 (85)	14 (93)	0.623
Ciprofloxacin non-susceptible	154 (92.2)	97 (92)	43 (93)	14 (93)	1.000
Faecal culture positive	38/158 (24.1)	21/101 (21)	15/42 (36)	2/15 (13)	0.123
Quantitative bone marrow counts, median (IQR)					
Untreated bone marrow (cfu/mL)	11 (3–33)	14 (4–44)	5 (2–28)	5 (1–18)	0.092
Gentamicin-treated bone marrow (cfu/mL)	4 (1–22)	6 (2–22)	2 (0–23)	2 (0–13)	0.045
Lysed bone marrow (cfu/mL)	14 (4–55)	20 (5–72)	11 (3–51)	7 (1–31)	0.087
Extracellular (cfu/mL)	2.5 (0–10)	4.0 (0–12)	2.0 (0–7)	1.5 (0–3)	0.254
Intracellular (cfu/mL)	10.5 (2–42)	14.0 (3–50)	5.0 (1–38)	5.0 (1–23)	0.054
Proportion of bacteria/cell	1.3 (0.6–2.5)	1.3 (1.0–2.6)	1.0 (0.2–2.0)	1.3 (0.5–2.0)	0.287
Proportion of bacteria intracellular (%)	80.8	77.8	71.4	76.9	

a Comparison of different age groups by chi square, Fisher's exact or Mann-Whitney U test as appropriate.

Twenty-four (14.4%) of the 167 culture-positive patients who had a quantitative bone marrow aspirate count were assessed as having severe disease at presentation or during the course of the study. Of the 24 patients, there were 16 (9.6%) with hepatitis, 12 (7.2%) with gastrointestinal bleeding, 3 (1.8%) with myocarditis, 3 (1.8%) with cholecystitis and 2 (1.2%) with encephalopathy. The clinical features, laboratory results and quantitative bone marrow counts of the patients with severe and non-severe disease are presented in Table 4. Patients with severe disease were more likely to present with abdominal pain and vomiting and hepatosplenomegaly. The counts for the untreated bone marrow, gentamicin-treated sample and lysed sample gave significantly higher values in patients with severe disease. The calculated extracellular count and number of bacteria per cell did not vary significantly according to the presence of disease severity, but there were significantly higher median counts of intracellular bacteria in severe disease compared with non-severe disease (46.0 vs 6.5 cfu/mL; p=0.005).

**Table 4.** The demographics and admission clinical features, laboratory results and quantitative bone marrow aspirate counts of 167 blood and/or bone marrow aspirate culture–confirmed typhoid fever patients who had a quantitative bone marrow aspirate bacterial count performed, categorised by disease severity

Covariate	All patients (N=167)	Severe (n=24)	Non-severe (n=143)	p-Value^a^
Male, n (%)	73 (44)	15 (63)	58 (41)	0.075
Age (years), median (IQR)	9 (6–12)	9 (7–13)	9 (6–12)	0.757
Days ill prior to admission, median (IQR)	7 (5–10)	7 (5–10)	7 (5–10)	0.645
Signs and symptoms, n (%)				
Abdominal pain	119 (71)	22 (91.7)	97 (67.8)	0.015
Constipation	107 (64)	16 (66.7)	91 (63.6)	0.823
Diarrhoea	137 (82)	22 (91.7)	115 (80.4)	0.255
Vomiting	79 (47)	17 (70.8)	62 (43.4)	0.015
Cough	52 (31)	7 (29.2)	45 (31.5)	1.000
Headache	103 (61)	15 (62.5)	88 (61.5)	1.000
Temperature ≥40°C	67 (40)	13 (54.2)	54 (37.8)	0.176
Hepatomegaly	107 (64)	22 (91.7)	85 (59.4)	0.002
Splenomegaly	13 (8)	5 (20.8)	8 (5.6)	0.023
Laboratory, median (IQR)				
Haematocrit (%)	34 (31–36)	33 (30–34)	34 (31–36)	0.092
White cell count (×10^9^/L)	6.5 (5.4–8.2)	5.8 (4.7–7.9)	6.8 (5.6–8.5)	0.055
Platelet count (×10^9^/L)	176 (138–247)	168 (107–268)	176 (138–247)	0.336
Neutrophil count (×10^9^/L)	4.3 (3.3–5.4)	3.9 (2.9–5.6)	4.5 (3.5–5.4)	0.276
Lymphocyte count (×10^9^/L)	1.9 (1.2–2.6)	1.7 (1.2–2.0)	1.9 (1.3–2.6)	0.086
AST (IU/L)	116 (78–210)	419 (222–535)	103 (74–164)	<0.001
ALT (IU/L)	76 (51–135)	217 (101–429)	73 (51–106)	<0.001
Isolate, n (%)				
Fully susceptible organism	15 (9)	1 (4.2)	14 (9.8)	0.699
MDR	149 (89)	22 (91.7)	127 (88.8)	1.000
Ciprofloxacin non-susceptible	154 (92)	23 (95.8)	131 (91.6)	0.695
Stool culture positive	38/158 (24)	3/24 (12.5)	35/134 (26.1)	0.198
Quantitative bone marrow count, median (IQR)				
Untreated bone marrow (cfu/mL)	11 (3–33)	39 (8–111)	8 (3–28)	0.013
Gentamicin treated bone marrow (cfu/mL)	4 (1–22)	17 (6–69)	3 (0–19)	0.002
Lysed bone marrow (cfu/mL)	14 (4–55)	47 (10–160)	12 (4–48)	0.013
Extracellular (cfu/mL)	2.5 (0–10)	3.0 (0–36)	2.0 (0–10)	0.577
Intracellular (cfu/mL)	10.5 (2–42)	46.0 (9–105)	6.5 (2–34)	0.005
Proportion of bacteria/cell	1.3 (0.6–2.5)	1.5 (0.9–2.8)	1.3 (0.5–2.5)	0.536
Proportion of bacteria intracellular (%)	80.8	93.9	76.4	

a Comparison of different age groups by χ^2^, Fisher's exact or Wilcoxon rank sum test as appropriate.

The association of the bone marrow counts with selected clinical and laboratory parameters are presented in Table 5. Higher extracellular counts were associated with patients with lower white cell and neutrophil counts, elevated AST and a positive blood culture. Higher intracellular counts were significantly associated with the presence of hepatomegaly, splenomegaly, elevated AST, a lower white cell and neutrophil count and a positive blood culture. For each of the treatment arms there was a significant correlation between the intracellular counts and fever clearance times. For extracellular counts there was a weak association in the patients treated with ofloxacin.

**Table 5.** Variables associated with the calculated extracellular and intracellular quantitative bone marrow aspirate bacterial counts

Variable	Events, n	Extracellular bone marrow ST load (cfu/mL), median (IQR)	Spearman's ρ	p-Value^a^	Intracellular bone marrow load (cfu/mL), median (IQR)	Spearman's ρ	p-Value^a^
Hepatomegaly	107	3.0 (0–12)		0.051	16.0 (3–52)		0.006
No hepatomegaly	60	1.0 (0–8)			5.0 (1–17)		
Splenomegaly	1	12.0 (1–40)		0.192	52.0 (31–76)		0.012
No splenomegaly	154	2.0 (0–10)			8.0 (2–36)		
Haematocrit (%)			−0.100	0.200		−0.109	0.164
White cell count (×10^9^/L)			−0.239	0.002		−0.243	0.002
Neutrophil count (×10^9^/L)			−0.208	0.007		−0.212	0.006
Lymphocytes (×10^9^/L)			−0.091	0.246		−0.124	0.110
Platelets (×10^9^/L)			−0.144	0.072		−0.143	0.073
AST (IU/L)			0.227	0.003		0.293	<0.001
ALT (IU/L)			0.116	0.137		0.148	0.058
MDR^c^	149	3.0 (0–12)		0.046	11.0 (2–46)		0.153
Non-MDR	18	1.5 (0–3)			5.5(0–15)		
Ciprofloxacin non-susceptible	154	3.0 (0–11)		0.338	11.0 (2–46)		0.142
Ciprofloxacin susceptible	13	2.0 (0–3)			2.0 (0–23)		
Positive blood culture		3.0 (0–12)		0.035	15.0 (2–50)		0.003
Negative blood culture		2.0 (0–3)			3.0 (1–8)		
Positive stool culture	38	2.0 (0–7)		0.899	7.0 (3–30)		0.254
Negative stool culture	120	3.0 (0–12)			13.5 (2–48)		
FCT^d^ with azithromycin treatment^e^	54		0.061	0.660		0.444	<0.001
FCT^d^ with ofloxacin treatment^f^	53		0.293	0.035		0.498	<0.001
FCT^d^ with ofloxacin and azithromycin treatment^g^	51		−0.334	0.465		0.489	<0.001
FCT^d^ with ceftriaxone and azithromycin treatment^h^	7		−0.334	0.465		0.321	0.482

^a^By Wilcoxon rank sum test.

^b^Correlation of continuous variable with viable ST load was assessed using Spearman's rank correlation.

^c^Resistant to chloramphenicol, amoxycillin and trimethoprim-sulphamethoxazole.

^d^Fever clearance time (FCT): time from the start of treatment until the body temperature reached 37.5°C and remained at 37.5°C for 48 h.

^e^Azithromycin suspension 10 mg/kg/d orally once a day (maximum 500 mg/d) for 7 d (tablets were used for adults).

^f^Ofloxacin 20 mg/kg/d orally in two divided doses (maximum 400 mg twice daily) for 7 d.

^g^Ofloxacin 15 mg/kg/d orally in two divided doses (maximum 300 mg twice daily) for 7 d combined with azithromycin suspension 10 mg/kg/d orally once per day (maximum 500 mg/d) for the first 3 d.

^h^Ceftriaxone 50–75 mg/kg/d intravenous once a day for 10 d combined with azithromycin suspension 10 mg/kg/d orally once per day (maximum 500 mg/d) for the first 3 d.

Extracellular and intracellular bone marrow aspirate counts were included with age, sex, duration of illness prior to admission to the hospital, the presence of an MDR phenotype and non-susceptibility to ciprofloxacin in a logistic regression model of associations with severe typhoid fever. The only factor independently associated with severe disease was the intracellular bacterial count (adjusted odds ratio 1.010 [95% CI 1.003 to 1.016]; p=0.006).

## Discussion

This study confirms and extends our previous findings concerning quantitative counts of bacteria in the bone marrow in typhoid fever.^25^ In the previous study, the median count in the bone marrow of 81 patients with uncomplicated typhoid was 9 cfu/mL (IQR 1–85). In this study, the result in 143 patients with uncomplicated typhoid was 11 cfu/mL (IQR 3–33). The study provides new evidence of significantly higher median counts of 39 cfu/mL (IQR 8–111) in patients with severe disease compared with 8 cfu/mL (IQR 3–28) in non-severe infection. The logistic regression model suggested an independent and significant association of the intracellular count and disease severity, although the effect size was very small and may not be biologically or clinically important. These results are also in keeping with the observations of Hoffman, who described the proportion of patients with a positive bone marrow culture in different severities of typhoid.^26^ In that study, 392 patients with typhoid fever had an admission BMC performed: 90% of cultures were positive in the 29 patients who died,75% were positive in the 90 severe typhoid patients who lived; and 56% were positive in the 273 non-severe typhoid patients. Higher counts of bacteria in the blood have been shown to correlate with severity in other Gram-negative bacteraemias,^23^ and a higher number of positive blood cultures correlated with 1-y mortality in non-typhoidal salmonellosis.^24^

We know little about the processes occurring at the sites of replication in the bone marrow and other reticuloendothelial tissues in typhoid fever. In this study we confirm the predominantly intracellular location of bacteria in the bone marrow with a calculated median number of intracellular bacteria of 10.5 cfu/mL (IQR 2–42). The number was significantly greater in those with severe disease (46 cfu/mL [IQR 9–105]) compared with those with non-severe infection (6.5 cfu/mL [IQR 2–34]; p=0.005). The calculated median number of extracellular bacteria was 2.5 cfu/mL (IQR 0–10), with no difference between severe and non-severe infection. Of note, there was no difference in the median proportion of bacteria per cell (1.3 [IQR 0.6–2.5]) across the clinical spectrum of disease despite the marked variation in the overall numbers of bacteria. This is consistent with data from a study using multicolour fluorescence microscopy in a mouse model of invasive *Salmonella* infection that showed the number of bacteria per phagocyte, counted by microscopy, followed a Poisson distribution with a mean of 1.89±0.12 (n=136).^30^ This study suggested that the growth of *Salmonella* in the liver resulted from the spread of bacteria to new infection foci rather that multiplication to high numbers in individual cells at the site of the initial focus.

Higher calculated intracellular bone marrow counts were associated with the presence of hepatomegaly, splenomegaly and elevated AST levels. This may be consistent with the increasing number of infection foci in the bone marrow reflecting a process that is also occurring in the liver and spleen. Higher calculated intracellular bone marrow counts were also associated with lower white cell and neutrophil counts, perhaps suggesting bone marrow suppression of neutrophil production. A limitation of this study is that we did not assess the quality or the histology of the bone marrow aspirate to link quantitative counts with the histological changes.

Elevated intracellular bone marrow counts were significantly associated with a prolonged fever clearance time in all treatment arms. For extracellular bone marrow counts, the same association was only weakly present with ofloxacin. The fever clearance time was correlated positively with bone marrow counts in our previous study with patients treated with 5 d of ofloxacin.^25^ This suggests that the bone marrow counts, in particular the intracellular counts, reflect the bacterial load in typhoid fever. There was no clear relationship between counts and antimicrobial resistance or faecal culture positivity, in contrast with our previous observations.^18,25^

In patients with confirmed typhoid fever, indicated by a positive culture from blood, bone marrow aspirate or faeces, 80% had a positive blood culture. This is a higher proportion than in a systematic review of previous studies, which reported a blood culture sensitivity of 61% (95% CI 52 to 70).^14^ Differences in the inoculated blood volume, the prevalence of prior antibiotic treatment and laboratory methods may contribute to this difference. We did not use a commercial automated blood culture system, which might be expected to further improve the culture yield. Consistent with previous studies, the proportion of patients with a positive blood culture declined from 86% in the first week of symptoms to 56% in those with symptoms for >2 weeks, whereas the proportion of bone marrow–positive samples remained between 89% and 94%.^4^

It is possible that pre-hospital use of antimicrobials, more likely with a delayed presentation but which we were unable to quantify in this study, may have been able to sterilise the blood but not the higher number of intracellular organisms in the bone marrow.

Limitations of the study include the lack of accurate information about pre-hospital treatment, the volumes of blood used for culture and the small numbers of patients with severe infection and none with intestinal perforation. The bone marrow was not collected at the same time as the blood culture, but invariably within 24–48 h. We think this delay is unlikely to have significantly influenced the results. The reporting of the findings of this study, performed between 1998 and 2002, has been delayed. Current treatment-seeking behaviour and antimicrobial susceptibility patterns may have changed over time and may also vary in other locations. We suggest the results are still relevant and reflect underlying processes in the biology of typhoid fever.

In this study we demonstrated a moderate additional yield of culture-positive cases from bone marrow compared with blood culture for diagnosis in patients with suspected typhoid fever. Elevated bacterial counts in bone marrow, particularly intracellular counts, were significantly associated with severe disease and prolonged fever clearance times with treatment with azithromycin, ofloxacin or a combination of the two. Intracellular bone marrow bacterial counts may reflect the total bacterial load in typhoid fever and may be an important factor in the development of severe disease.

## Data Availability

The data from this study are available upon request to the corresponding author
